# Ethyl Cellulose and Hydroxypropyl Methyl Cellulose Blended Methotrexate-Loaded Transdermal Patches: In Vitro and Ex Vivo

**DOI:** 10.3390/polym13203455

**Published:** 2021-10-09

**Authors:** Muhammad Shahid Latif, Abul Kalam Azad, Asif Nawaz, Sheikh Abdur Rashid, Md. Habibur Rahman, Suliman Y. Al Omar, Simona G. Bungau, Lotfi Aleya, Mohamed M. Abdel-Daim

**Affiliations:** 1 Department of Pharmaceutics, Faculty of Pharmacy, Gomal University, Dera Ismail Khan 29050, Pakistan; shahidlatif1710@gmail.com (M.S.L.); asifnawaz676@gmail.com (A.N.); sheikhabdurra-shid11@gmail.com (S.A.R.); 2 Advanced Drug Delivery Laboratory, Pharmaceutical Technology Department, Faculty of Pharmacy, International Islamic University Malaysia, Kuantan 25200, Pahang, Malaysia; 3 Department of Global Medical Science, Wonju College of Medicine, Yonsei University, Seoul 26426, Gangwon-do, Korea; pharmacisthabib@gmail.com; 4 Department of Zoology, College of Science, King Saud University, P.O. Box 2455, Riyadh 11451, Saudi Arabia; syalomar@ksu.edu.sa; 5 Department of Pharmacy, Faculty of Medicine and Pharmacy, University of Oradea, 410087 Oradea, Romania; simonabungau@gmail.com; 6 Chrono-Environnement Laboratory, UMR CNRS 6249, Bourgogne, Franche-Comté University, CEDEX, F-25030 Besançon, France; lotfi.aleya@univ-fcomte.fr; 7 Pharmacology Department, Faculty of Veterinary Medicine, Suez Canal University, Ismailia 41522, Egypt

**Keywords:** transdermal drug delivery system (TDDS), hydroxypropyl methyl cellulose (HPMC), ethyl cellulose (EC), methotrexate, patches

## Abstract

Transdermal drug delivery systems (TDDSs) have become innovative, fascinating drug delivery methods intended for skin application to achieve systemic effects. TDDSs overcome the drawbacks associated with oral and parenteral routes of drug administration. The current investigation aimed to design, evaluate and optimize methotrexate (MTX)-loaded transdermal-type patches having ethyl cellulose (EC) and hydroxypropyl methyl cellulose (HPMC) at different concentrations for the local management of psoriasis. In vitro release and ex vivo permeation studies were carried out for the formulated patches. Various formulations (F1–F9) were developed using different concentrations of HPMC and EC. The F1 formulation having a 1:1 polymer concentration ratio served as the control formulation. ATR–FTIR analysis was performed to study drug–polymer interactions, and it was found that the drug and polymers were compatible with each other. The formulated patches were further investigated for their physicochemical parameters, in vitro release and ex vivo diffusion characteristics. Different parameters, such as surface pH, physical appearance, thickness, weight uniformity, percent moisture absorption, percent moisture loss, folding endurance, skin irritation, stability and drug content uniformity, were studied. From the hydrophilic mixture, it was observed that viscosity has a direct influence on drug release. Among all formulated patches, the F5 formulation exhibited 82.71% drug release in a sustained-release fashion and followed an anomalous non-Fickian diffusion. The permeation data of the F5 formulation exhibited about a 36.55% cumulative amount of percent drug permeated. The skin showed high retention for the F5 formulation (15.1%). The stability study indicated that all prepared formulations had very good stability for a period of 180 days. Therefore, it was concluded from the present study that methotrexate-loaded transdermal patches with EC and HPMC as polymers at different concentrations suit TDDSs ideally and improve patient compliance for the local management of psoriasis.

## 1. Introduction

Psoriasis is a chronic inflammatory disease affecting about 1–3% of the world’s population [1]. This lifelong disease has an equal gender distribution, and its incidence rate may vary from 50 to 140 new cases per 100,000 cases per year [2]. Its mortality risk is increased exponentially in terms of severe psoriasis when compared to the general population, even though it is usually not life threatening [3]. Psoriasis leads to decreased patient quality of life due to its link with high levels of morbidity and ailment. The management protocols of psoriasis vary depending upon the severity index of the disease [4].

Topical agents constitute first-line therapy, typically sufficient for active management of the disease to combat mild to moderate types of psoriasis [5]. Phototherapy and systemic management are crucial to consider when either suboptimal effects arise from topical therapies or when the intensity of the psoriasis limits the use of topical agents [6]. Currently available systemic tools comprise biological and nonbiological therapies, which are utilized as monotherapy or in combination with other modalities to manage moderate to severe psoriasis [7].

Methotrexate, orally administered retinoid, and cyclosporine represent prominent nonbiological systemic agents. Methotrexate administered via oral and parenteral routes presents an excellent therapeutic strategy to treat psoriasis owing to its epidermal cell proliferation inhibition, as well as anti-inflammatory actions at low doses [8]. However, a large number of reported toxicities due to methotrexate systemic administration, such as liver impairment, and gastric side effects, including diarrhea vomiting and stomatitis, appear [9]. Methotrexate is a folate antagonist, and it displays prominent antineoplastic activity, as well as having a use for psoriasis management [10]. It competitively inhibits the enzyme dihydrofolate reductase, leading to DNA inhibition synthesis. Methotrexate, when delivered to the psoriatic site by means of transdermal drug delivery, has the potential to reduce side effects associated with this drug and avoid first-pass metabolism [11]. A major problem with methotrexate is that the drug is hydrosoluble and available in ionized form at physiological pH (7.4), leading to limited capacity for passive diffusion [12].

To minimize the likelihood of side effects, as well as skin permeation, and to maintain a therapeutic concentration in the target tissues, numerous approaches have been proposed, such as liposomes, polymeric nanoparticles, microspheres, solid lipid nanoparticles, nanoemulsions and nanoemulsion gel and patch formulations [13]. However, transdermal patches offer numerous advantages in terms of ease of preparation, high loading capacity for hydrophilic and lipophilic drugs and long-term stability with improved dermal delivery [14]. A transdermal patch is used to deliver a specific dose of medication through the skin and into the bloodstream. Transdermal delivery provides controlled, consistent drug administration and produces continuous drug input. It has a short biological half-life and eliminates pulsed entry into the systemic circulation [15]. It is convenient and especially evident in patches that require application only once a week. Such a simple dosing regimen could enhance patient compliance with drug therapy [16].

Polymers are widely used in modern pharmaceutical technologies, and they play a vital role in drug delivery advancements. Polymers act as carriers in targeted therapies and offer controlled drug delivery while reducing the bitter taste of drugs [17]. Hydroxypropyl methylcellulose (HPMC) is a derivative of cellulose of hydrophilic nature [18]. It is widely used in controlled-release formulations due to its swelling, gelling and thickening properties. Furthermore, HPMC is nontoxic in nature, and its swelling and easy compression properties make it convenient for use in the preparation of controlled drug delivery systems [19].

Ethylcellulose (EC) is a derivative of cellulose of hydrophobic nature. It is a white to light free-flowing powder used widely in the manufacturing of controlled drug delivery systems. EC has very limited side effects; hence, it is considered safe to employ in tablets, oral capsules, ocular or vaginal preparations and topical preparations [20]. EC is an inert, hydrophobic polymer that exhibits certain properties such as good stability during storage, lack of toxicities and good compressibility, which are suitable for designing controlled drug delivery systems [21].

This study was undertaken to develop methotrexate-loaded matrix-type patches by employing a combination of hydrophilic and hydrophobic polymers, hydroxypropyl methylcellulose (HPMC) and ethyl cellulose (EC), for the pertinent and effective dermal treatment of psoriasis, with improved cutaneous deposition of methotrexate to enhance its local effect. The success associated with methotrexate through dermal application via patch formulations could also be represented by increased patient compliance due to the topical administration of therapeutic substances, representing a less invasive and more comfortable and convenient route of administration.

## 2. Materials and Methods

### 2.1. Materials

Methotrexate was kindly gifted by Wilsons Pharmaceutical (Pvt.) Ltd., Pakistan. Ethyl cellulose (EC) and hydroxypropyl methyl cellulose (HPMC) (Dow Chemical Company, 693 Washington St, #627, Midland, MI, 48640, USA, +1-989-636-1000) were used as rate-controlling polymers. Ethanol (Sigma-Aldrich Inc., P.O. Box 14508, St. Louis, MO, 63178, USA, +1-314-771-5750), PEG-400 (Sigma-Aldrich Co., 3050 Spruce Street, St. Louis, MO, 63103, USA, +1-314-771-5765), sodium hydroxide (NaOH) (Sigma-Aldrich Chemie GmbH, Riedstrasse 2, 89555 Steinheim, Germany, +49-7329-970), dichloromethane and calcium chloride (Dow Chemical Co., 2030 Dow Ctr, Midland, MI, 48674, USA, +1-989-638-8173) were used in the preparation of patches and buffers. Analytical-grade chemicals were used in this study.

### 2.2. Preparation of Transdermal Patch

The solvent evaporation technique was used for the formulation of methotrexate-loaded patches, with EC and HPMC as rate-controlling polymers at different concentrations (Table 1). The polymers were weighted accurately using an analytical weighing balance (Shimadzu AX 200, Kyoto, Japan), placed in a solvent system (15 mL) comprising ethanol and dichloromethane (1:1) and allowed to swell for 6 h. The plasticizer used was PEG-400. A 100 µL volume of ethanolic hydrochloric acid was taken in a beaker, and a proper amount of methotrexate was added. Dichloromethane and ethanol (1:1) were taken in a separate beaker and placed in a sonicator (Elma D-78224, Germany) for 2 min. The drug and polymers were mixed homogeneously by slow stirring. A uniform dispersion was poured in Petri dishes with an area of 19.5 cm^2^. The Petri dishes were placed in an oven (Memmert, Germany) at 40 °C for 12 h.

### 2.3. ATR–FTIR Analysis (Preformulation Study)

ATR–FTIR analysis was carried out on the pure drug (methotrexate) and various physical mixtures of patch formulations (F1 to F9) to investigate possible interactions. A total of 32 scans were observed for each spectrum at a resolution of 4 cm^−1^ from 4000 to 600 cm^−1^.

### 2.4. Physicochemical Evaluation of Patches

The physicochemical properties of the formulated patches were evaluated using the following parameters.

#### 2.4.1. Surface pH

The surface pH of the formulated patches was evaluated by placing a 1 cm^2^ portion of a patch in 1 mL of distilled water for 2 h at room temperature (25 ± 2 °C) in a test tube. Excess water from the test tube was removed by the filtration process. A pH meter (InoLab^®^, Xylem Analytics, Dr. Karl Slevogt Street 1. Weilheim 82362, Germany) was used for the identification of the surface pH of the formulated patches. The pH meter was placed at the swollen part of the patch, and three readings were recorded for the average (mean ± SD) result [22].

#### 2.4.2. Physical Appearance

All formulated patches were physically inspected for smoothness, color, clarity, transparency, and homogeneity.

#### 2.4.3. Thickness

The uniformity of thickness was evaluated for all formulated patches. A vernier caliper (Germany) was used for the evaluation of the thickness of the formulated patches. The thickness of patches was evaluated at 6 different places, and then the average was calculated [23].

#### 2.4.4. Weight Uniformity

All formulated patches were weighed individually for weight uniformity. An analytical weighing balance (Shimadzu AX 200, Kyoto, Japan) was used for the determination of weight. Individual weight was compared with average weight [24].

#### 2.4.5. Folding Endurance

The efficacy of the plasticizer was investigated with the folding endurance test. The folding of a patch at the same point until a break or crack appears shows the folding endurance capacity of a patch. At the same point, a patch was folded several times without cracking or breaking defines the value of folding endurance. The folding endurance test was conducted for all formulated patches [24].

#### 2.4.6. Moisture Uptake

The formulated patches were weighed accurately for the determination of percent moisture uptake. Aluminum chloride and the patches were placed in a desiccator to maintain humid conditions. After 3 days, the patches were taken out of the desiccator. The patches were weighed again. The difference between the initial and final weights of the patches gave the value of percent moisture uptake. Finally, average percent moisture uptake was calculated [25].

% Moisture Uptake = (wf − wi)/wi × 100(1)

where wf is the final patch weight, and wi is the initial patch weight.

#### 2.4.7. Moisture Loss

All formulated patches were weighed individually for the determination of moisture loss. The patches, along with anhydrous calcium chloride, were placed in the desiccator at 37 °C in order to maintain dry conditions. After 3 days, the patches were taken out of the desiccator. The patches were weighed again. The difference between the initial and final weights of the patches gave the value of percent moisture loss. Finally, average percent moisture loss was calculated [14].

% Moisture Loss = (wi − wf)/wi × 100(2)

where wi is the initial weight, and wf is the final weight.

#### 2.4.8. Moisture Content

The formulated patches were weighed accurately for the determination of moisture content. The patches, along with silica, were placed in the desiccator at room temperature for 24 h. The patches were taken out of the desiccator and weighed again until a constant weight was calculated. Percent moisture content was calculated using the following equation [26].

% Moisture Loss = (wi − wf)/wi × 100(3)

whereas wi is the initial patch weight, and wf is the final patch weight

#### 2.4.9. Tensile Strength and Percent Elongation at Break

The mechanical properties of the formulated patches were determined using a pulley system. A scale was used for the identification of the initial patch length. One end of the patch was tied with a thread, while the second end was tied with a rope crossing over the pulley. A weighing pan was attached to the hanging side of the thread. Gradually, weight was added until a crack or break appeared in the patch. The total weight in the pan was calculated for the tensile strength. The thread pointer indicated percent elongation of work. From the following equation, the total amount of force (tensile strength, kg/cm^2^) required to break a patch was calculated.

Tensile Strength = F/(a.b(1+L/I))(4)

where,

F is the force needed to break a patch, a is the patch width (cm) and b is patch thickness (cm).

L is patch length (cm), and I is patch elongation before patch breakage (cm). The percent elongation of the patches was determined from the following equation [27].

% Elongation = (Lf − Li)/Li × 100(5)

where Lf is the final patch length before breaking, and Li is the initial patch length.

#### 2.4.10. Drug Content Uniformity

Drug content uniformity was evaluated for the formulated patches. A patch was placed in a volumetric flask filled with phosphate buffer (pH = 7.4) and then placed in a sonicator for 8 h. After sonication, the solutions were filtered. A double-beam UV–visible spectrophotometer (Shimadzu 1601, Kyoto, Japan) was used for the identification of drug content using a 303 nm wavelength [11].

#### 2.4.11. Water Vapor Transmission Rate

Oven-dried and properly washed equal-diameter glass vials were used as transmission cells. In the transmission cells, 1 g of anhydrous calcium chloride was kept. At brim, the formulated patches were fixed. Transmission cells were weighed and then placed in the desiccator. Potassium chloride solution was kept closed in the desiccator to maintain 84% humidity. After predetermined time intervals, i.e., 6, 12, 24, 36, 48 and 72 h, cells were removed from the desiccator. Then, the cells were weighed again for the identification of the water vapor transmission rate [28].

### 2.5. Stability Studies

Stability studies were carried out for a period of 180 days for all formulated patches. The incubator was maintained at 37 ± 0.05 °C and 75 ± 5 RH, and the patches were kept for stability studies. After a regular interval of 30 days, drug content and physical appearance were evaluated for the formulated patches. Drug content determination was carried out according to the procedure described in Section 2.4.1.

### 2.6. Skin Irritation Studies

For skin irritation studies, healthy male albino rabbits (2–2.5 kg) were used, and proper NOC was taken from the Research Ethical Review Board of Gomal Center of Pharmaceutical Sciences, Faculty of Pharmacy, Gomal University, Dera Ismail Khan, KPK, Pakistan. The rabbits used in this study were given standard food at least three days before administration of the formulations. The standard food was prepared according to a published recipe composed of 10% white fish meat, 18% middlings, 20% grass meal and 40% bran [29]. Water was also allowed ad libitum. All rabbits were housed in a temperature (25 ± 2 °C)- and relative-humidity-controlled (50 ± 10%) room. The sparse hairs on the abdomen of each rabbit were carefully shaved a day before the scheduled experiment with an electrical clipper without damaging the stratum corneum. The application area was swept with dry cotton. The Draize patch test was used for skin irritation studies. Five groups, each containing 3 rabbits, were selected for skin irritation (hypersensitivity) reactions. Group 1 served as the nontreated group, and Group II served as the control group with USP adhesive tape. Group III was served with methotrexate-loaded patches, Group IV was served with 0.8% *v*/*v* aqueous solution of formalin (which is a standard irritant) and Group V was served with blank patches. The skin irritation study was carried out for a period of 1 week. A visual scoring scale was used for the identification of skin irritation grades. Skin irritation was graded as follows: “0” indicated no skin irritation, “1” indicated slight skin irritation, “2” indicated well-defined skin irritation, “3” indicated moderate-type skin irritation and “4” indicated scar formation on the skin [29].

### 2.7. In Vitro Drug Release Studies

In Vitro drug release studies were carried out using a Franz diffusion cell apparatus (model _γ_9-CB (71026), PermeGear, Hellertown, PA, USA). A Tuffryn membrane was used as a synthetic membrane for drug release from the formulated patch of methotrexate with EC and HPMC at different concentrations. Between the donor and receptors compartments, the Tuffryn membrane was placed. A 1 cm^2^ area of the formulated patch was placed over the Tuffryn membrane. The formulated patch piece was placed in such a manner that the drug-releasing surface faced the Tuffryn membrane. Phosphate buffer (pH = 5.5) was used in the receptor compartment. The receptor compartment is surrounded by water jackets in which water circulates. The receptor fluid temperature was maintained at 32 ± 0.5 °C. Magnetic beads were used for the stirring of receptor fluids. At predetermined time intervals of 0.5, 1, 1.5, 2, 4, 8, 12, 16, 20 and 24 h, a 2 mL sample was taken from the receptor fluid. In order to maintain sink conditions, fresh receptor fluid of an equal volume was added to the receptor compartment. For the determination of drug content, these samples were analyzed spectrophotometrically at a 303 nm wavelength [30].

#### Kinetic Model Profiling

The drug release data were fitted into a Korsmeyer–Peppas kinetic model to investigate the mechanism of drug release [31,32]. The power-law equation is shown below

Mt/M∞ = Kt^n (6)

where Mt and M∞ are the fractions of drug released after time t.

K represents the constant rate.

n represents the exponential release value.

When n = 0.5, it is a quasi-Fickian diffusion mechanism.

When n > 0.5, drug release occurred by an anomalous non-Fickian, Case II or zero-order release mechanism

When n = 0, it indicates a zero-order release mechanism.

### 2.8. Ex Vivo Permeation Studies

#### Preparation of Rabbit Skin

For skin irritation studies, healthy male albino rabbits (2–2.5 kg) were used. For ex vivo permeation studies, proper NOC was taken from the Research Ethical Review Board of Gomal Center of Pharmaceutical Sciences, Faculty of Pharmacy, Gomal University, Dera Ismail Khan. The rabbits were allowed to roam freely and take food (standard) and water (ad libitum) at their own will. After administering an overdose of ketamine and xylazine injections, the rabbits were sacrificed. Hairs from the abdominal region of each rabbit were surgically removed. The skin was then dipped in warm water at 60 °C for 45 s for adhering fats. Excised skin was placed in distilled water, washed at –20 °C and stored until further use [30].

For the determination of drug (methotrexate) permeation from the formulated patches across rabbit skin, a Franz diffusion apparatus was used for this study. Before starting the experiment, excised skin that was kept at −20 °C was hydrated for at least 1 h. Then, between the donor and receptor compartments, the skin was placed. The stratum corneum (SC) side of the skin was placed facing the donor compartment of the Franz cell apparatus. Then, a 1 cm^2^ piece of the formulated patch was placed over the rabbit skin. The drug-releasing surface of the formulated patch faced the SC of the rabbit skin. In the receptor compartment, phosphate buffer (pH 7.4) was used. The temperature of the receptor fluid was maintained at 37 ± 0.5 °C by means of water circulating in the water jackets around the receptor compartment. The receptor fluids were stirred by means of magnetic beads. At predetermined time intervals of 0.5, 1, 1.5, 2, 4, 8, 12, 16, 20 and 24 h, a 2 mL sample was taken from the receptor fluid. In order to maintain sink conditions, fresh receptor fluid of an equal volume was added to the receptor compartment. For the determination of drug content, these samples were analyzed spectrophotometrically at a 303 nm wavelength [30].

### 2.9. Drug Retention Study

The drug retention study was carried out after the permeation experiment. Skin from the Franz cell apparatus was removed carefully and cleaned with phosphate buffer solution, then dried and cut into small pieces. The skin pieces were then stirred in phosphate buffer (pH = 7.4) overnight. Retained drug from the skin was extracted using methanol, and samples were centrifuged. The supernatant was filtered through a 0.45 µm cellulose acetate filter. The filtrate was analyzed with a UV–visible spectrophotometer at a 303 nm wavelength.

### 2.10. Statistical Analysis

One-way ANOVA was used as the statistical tool. A value of *p* < 0.05 was considered significant. All experiments were performed in triplicate, and the result was expressed as the mean value ± standard deviation.

## 3. Results

TDDS is user friendly, painless, and convenient, and it usually leads to enhanced patient compliance. Transdermal patches control drug delivery by employing different combinations of polymers. In the current study, various polymers were used to prepare methotrexate-loaded transdermal patches. The polymers used were ethyl cellulose (EC) and hydroxypropyl methyl cellulose (HPMC) at different ratios.

### 3.1. Drug Excipient Compatibility Studies (ATR–FTIR Analysis)

The methotrexate spectrum was compared with the spectrum of EC and HPMC polymers formulations at different concentrations.

**Figure 1:**
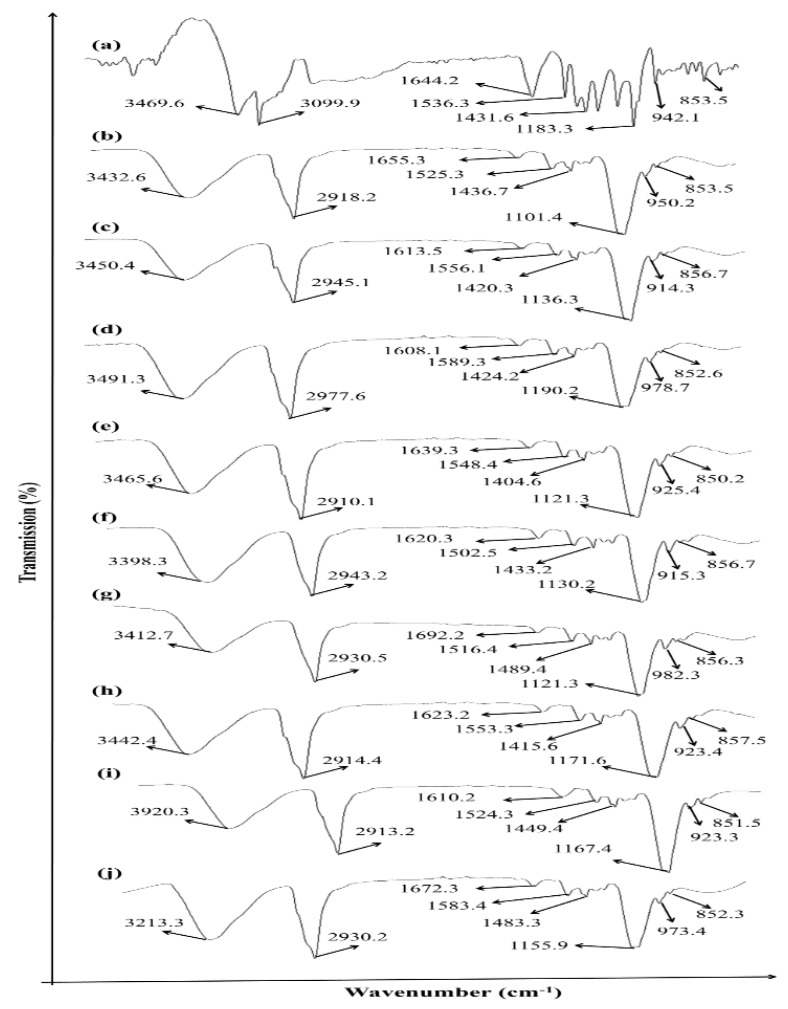
ATR–FTIR spectra: (**a**) MTX; (**b**) F1; (**c**) F2; (**d**) F3; (**e**) F4; (**f**) F5; (**g**) F6; (**h**) F7; (**i**) F8; (**j**) F9.

The methotrexate ATR–FTIR spectrum shows its characteristic absorption band as a broad signal at 3450 cm^−1^ (O–H being stretched from the carboxyl group overlaying with O–H being stretched from crystallized water). At 3080 cm^−1^ (primary amine, N–H stretched), 1670–1600 cm^−1^ is allocated to C=O stretching (–C=O stretched from the carboxylic group and C=O stretched from the amidic group (Figure 1). Hence, the C=O band is split into a double in the methotrexate sample). The corresponding N-H band from the amidic group appears in the spectral range of 1550–1500 cm^−1^. It is partly overlapped with the aromatic C=C stretching. The carboxylic group band appears in the range of 1400–1200 cm^−1^, corresponding to –C–O stretching. The molecular structure of the entire formulated patch of methotrexate indicates that it is in good agreement, which confirms its purity. The ATR–FTIR spectra of the physical mixture of methotrexate and polymers show major peaks. The FTIR spectrum of methotrexate with EC and HPMC polymers at different concentrations was compared with the methotrexate spectra. The major peaks of methotrexate and polymers were found preserved.

### 3.2. Physicochemical Assessment of Methotrexate-Loaded Transdermal Patches

All fabricated patch formulations of methotrexate were tested for physicochemical characterization. The results of various physicochemical tests revealed that all formulated patches were clear, smooth, transparent, flexible and nonsticky in appearance. The surface pH of all formulated patches (F1–F9) was found to be within the acceptable range, i.e., 5–5.9. Hence, skin irritation did not occur. Patch (F1–F9) thickness ranged between 0.50 and 0.60 nm, which showed uniform thickness. The weight ranged between 72.25 ± 0.08 and 78.67 ± 0.004 mg, which showed that the formulated patch weights were almost similar (Table 2). All formulated patches (F1–F9) passed the folding endurance test. The folding endurance of all formulated patches was more than the predefined range of folding endurance, i.e., ≥30. Hence, all formulated patches proved to be the best and acceptable dosage forms used transdermally. However, there was an increase in moisture content with an increase in hydrophilic polymers. This may be due to the higher affinity of water for hydrophilic polymers than hydrophobic polymers. The percent moisture uptake was found to be higher in the patch containing EC because it absorbs moisture. The low moisture content in the prepared formulations helped them to remain stable and free from being completely dry and brittle patches. The moisture loss ranged from 6.8 ± 0.38 to 8.28 ± 0.85. Similarly, the moisture absorption observed was found to be satisfactory and ranged from 8.45 ± 1.22 to 12.79 ± 1.46. The tensile strength and elongation values of the formulated patches ranged between 9.36 kg/cm^2^ and 12.75 kg/cm^2^, and proper flexibility was observed, as indicated by the high values. All formulated patches showed uniformity in drug content that was quite good and ranged between 97.42% and 99.13%. The results of this study show that the formulated patches could produce transdermal matrix-type patches with uniform drug contents. The plasticizer used was PEG-400 to reduce the brittleness of the patches. The current study indicates that the addition of PEG-400 at 25% *w/w* of polymers produces uniform, flexible and smooth patches. Patches formulated with the addition of PEG-400 as plasticizer were found to be best for tensile strength and folding endurance properties.

### 3.3. Stability Studies

**Figure 2:**
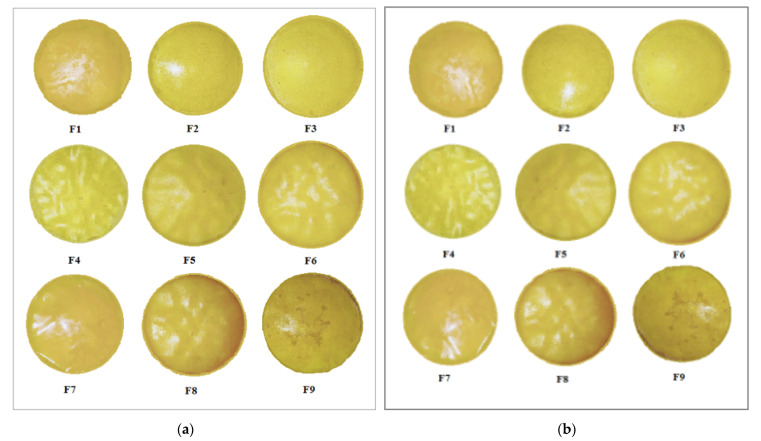
Physical appearance of formulated (F1–F9) patches shown at Day 1 (**a**) and after 180 days (**b**).

Stability studies of all formulated patches are shown in Table 3. All formulated patches showed almost similar drug content data, as observed at the beginning of the study. All formulated patches showed acceptable, flexible and elasticity properties at the beginning Figure 2a, and end of this study in Figure 2b, thus ensuring the stability of the formulated patches.

### 3.4. Skin Irritation Study

The skin irritation study revealed that no irritation erythema or edema occurred. During the study period and after the removal of transdermal patches, no edema or erythema was found, which indicates that the formulations were nonirritant, while formalin (standard irritant) produced severe erythema and edema. The Draize test was negative, indicating that no skin irritation occurred if the score of the tests was less than 2 showed in Table 4.

### 3.5. In Vitro Drug Release Study

**Figure 3:**
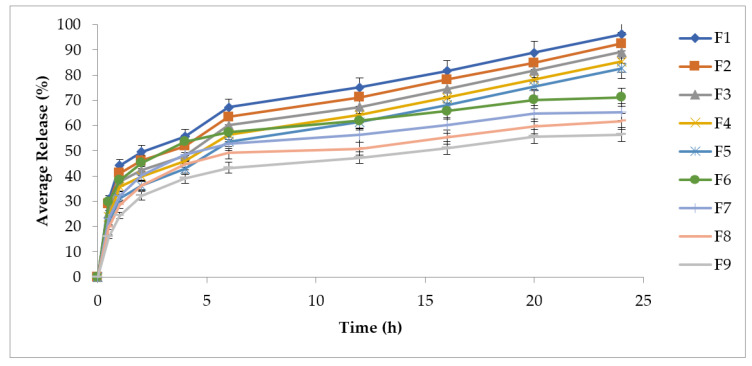
Release profile of MTX from F1 to F9, data were expressed as mean ± SD, *n* = 3.

In vitro drug release studies are needed for predicting the reproducibility of the rate and duration of drug release. The results from the in vitro drug release studies show that the release from the formulated patches increases with an increase in the concentration of the hydrophilic polymer (HPMC). The formulations from F1 to F5 showed higher release, while formulations from F6 to F9 showed lower release over a time period of 24 h in Figure 3. Hence, from the drug release profile of all formulations, F5 showed the best controlled-release profile of 82.71%. This may be attributed to the presence of the hydrophobic polymer (EC) and hydrophilic polymer (HPMC) in the ratio of 1:5. It was noted that the hydrophilic polymer released the drug at a faster rate than the hydrophobic polymer. The F5 formulation is an optimized formulation. The diffusion mechanism is responsible for drug release for transdermal drug delivery systems. This mechanism involves drug transport from the polymer matrix into the respective medium based on the concentration gradient. The variation in the concentration gradient leads to drug release, as well as a greater distance for diffusion. This could be the most probable reason for the comparatively slower rate of drug diffusion when the distance for diffusion increases.

### 3.6. Drug Release Kinetics

All the data obtained from the formulated patches were fitted to the Korsmeyer–Peppas model for the confirmation of the exact drug release behavior showed in Table 5. In our present study, the F5 formulation showed the best fit with the Korsmeyer–Peppas equation (R^2^ = 0.974), showing an anomalous or non-Fickian diffusion mechanism of drug release (*n* = 0.50131). There was complete and controlled drug release of methotrexate found over a period of 24 h. The optimized formulation for the current study was F5. Thus, F5 releases the drug at the predefined rate for a prolonged period of time into the systemic circulation, leading to minimal dose frequency and adverse effects.

### 3.7. Ex Vivo Drug Permeation Study

**Figure 4:**
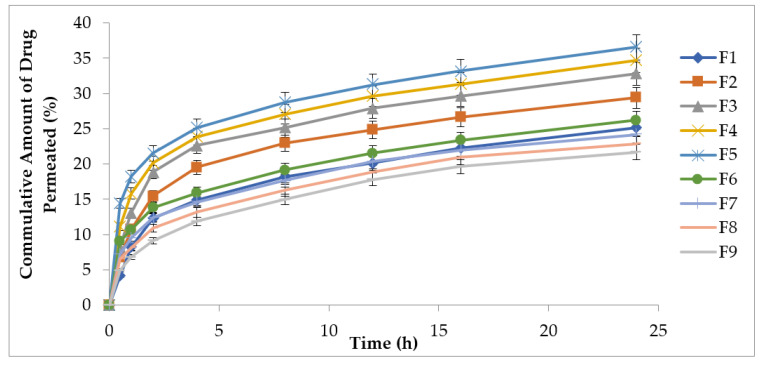
Percent cumulative amount of MTX permeated (F1–F9), Data were expressed as mean ± SD, *n* = 3. Significant compared to formalin (*p* < 0.05).

The ex vivo permeation result of methotrexate-loaded patches having EC and HPMC at different concentrations showed in Figure 4. The F5 formulation exhibited a maximum percent cumulative amount of drug permeation (34.68%), producing a significant difference (*p* < 0.05) compared to the F1 formulation (25.11%) in 24 h. The F1 formulation produced 9.14 µg/h/cm^2^, which is less than the required flux of 20.11 µg/h/cm^2^. An increase in HPMC concentration increases flux values; thus, the F5 formulation, containing a greater amount of HPMC, produced about 1.5-fold greater flux compared to the target flux. Similarly, the F9 formulation exhibited a cumulative drug permeation of 21.68% compared to the F1 formulation, which exhibited a cumulative drug permeation of 25.11%. The flux value of F9 was found to be 9.23 µg/h/cm^2^.

### 3.8. Drug Retention Analysis

**Figure 5:**
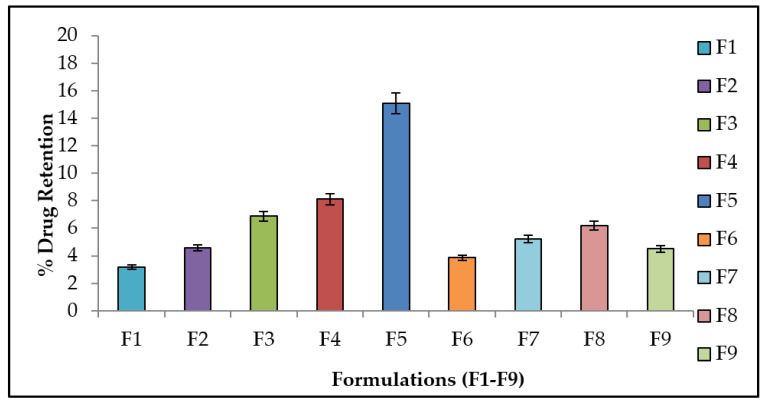
Skin drug retention analysis of methotrexate patches (F1–F9), Data were expressed as mean ± SD, *n* = 3. Significant compared to formalin (*p* < 0.05).

The drug retention study (see Figure 5), revealed that the retention of methotrexate is more in the deep layers of the skin. In the case of F5, when compared to other formulations, there is a statistically significant difference in drug retention primarily in the epidermis and dermis (ANOVA, *p* < 0.05).

## 4. Discussion

Preformulation studies play a very crucial role in successfully creating a formulation. In order to determine drug excipient compatibility, ATR–FTIR analysis was carried out. ATR–FTIR is a nondestructive and quick technique used for obtaining the IR spectrum of a pure drug (methotrexate), as well as various patch formulations (F1 to F9). It is used for the identification and characterization of interactions between drugs and synthetic, semisynthetic, and native macromolecules. The ATR–FTIR spectra revealed several peaks in the final formulation, which confirmed the chemical structure retained within the drug with efficient loading into the formulation. The current study showed no chemical interaction between methotrexate and the physical mixtures with polymers (EC, HPMC) used [33]. The solvent evaporation technique was employed for the formulation of methotrexate-loaded transdermal-type patches having HPMC and EC at different concentrations [34]. The surface pH of the formulated patches was determined to investigate the possibilities of irritation during in vivo studies. This test is of utmost importance during transdermal drug delivery because alkaline or acidic pH causes irritation to the skin [35]. Uniform weight measurements and thicknesses were observed, which were evident due to low standard deviation values [26]. The folding endurance value is important during the formulation of transdermal patches. This test indicates that formulated patches can integrate with skin folding and do not break during use [24]. The drug release pattern of transdermal matrix-type patches can be affected by moisture uptake and moisture loss. In this study, the values of moisture content and moisture uptake were low. This indicates that the patches remain stable during long-term storage, and brittleness is reduced. Formulated patches are protected from microbial contaminations, having low moisture uptake and reduced bulkiness [8]. Tensile strength and elongation are related to the effectiveness of the plasticizer used in formulations [36]. It is necessary for the drug distribution to be homogenous and uniform because it helps in the evaluation of sustained drug deliveries from formulated patches. Uniform drug content data were observed from the formulated patches, which were revealed by low standard deviation values. Plasticizer use in the formulation of transdermal patches is necessary to improve the patch-forming properties and physical appearance of the patches. This prevents the patches from cracking and breaking and increases patch flexibility for obtaining the desired mechanical properties [37]. The successfully formulated patch formulations were screened for their irritation potential, as withdrawal of the patch from the site of application resulted in irritation in the form of erythema and edema, so it is necessary for patch formulations to completely lack irritation potential in order to obtain patient acceptability and a therapeutic outcome [3]. The in vitro drug release profile of the methotrexate-loaded patches showed an initial burst release, followed by a gradually approaching plateau, giving an indication of the controlled-release behavior of the matrix formulations. This burst release might be due to the release of superficially adhered drug contents. Drug release could be prolonged by adjusting adequate ratios of EC and HPMC [38]. There was a decrease in the release rate with an increasing concentration of HPMC in the formulations. This is because increased proportions of HPMC in the matrix resulted in a proportionate decrease in the amount of water uptake, leading to lesser drug release. EC, owing to its hydrophobic nature, produced a retarded drug release from the matrix [16]. The optimized F5 formulation showed a controlled-release pattern. This is advantageous in the case of chronic conditions such as psoriasis because rapid or burst release is not useful owing to its toxicity potential due to greater drug release and faster absorption of the drug in inflamed psoriatic lesions. The Korsmeyer–Peppas model was used for kinetic drug profiling. Data were fitted into this model in order to investigate the Fickian or non-Fickian diffusion pattern, followed by formulated patches. The value of “n” determines whether the drug release mechanism that weathers the release pattern is Fickian or non-Fickian diffusion. If the value of “n” is equal to 0.5, the diffusion is said to be Fickian. If the value of “n” ranges between 0.5 and 1, the diffusion is said to be anomalous diffusion. When the value of “n” is equal to 1, the diffusion is said to be Case II transport behavior [39]. The in vitro skin permeation profile is considered a significant tool to exclude the risks of unfortunate drug effects. In vitro skin permeation experiments describe the rate and mechanism of the percutaneous absorption of drugs. Studies have shown that diffusion rate is affected due to the physicochemical characteristics of the formulations. These include hydrogen bonding, drug loading capacity, surface charge and mode of application [16]. The skin permeation experiment concluded that patch formulations play a significant role in controlling methotrexate release, as well as drug targeting to the skin. Studies have shown that diffusion rate is affected due to the physicochemical characteristics of the formulation. During skin retention studies, greater retention was observed. This might be due to the strong interaction of the drug with keratinocytes. It is also expected that more drug is retained in the dermis compared to the epidermis, as revealed by skin anatomy studies, which show the dermis layer is thicker than that of the epidermis. Regardless, the accumulation of more amounts of drug in the deeper layer of skin is advantageous, as these layers are mainly affected by psoriasis [40]. Stability studies performed for the formulated patches were carried out for 180 days. After specific intervals of 30 days, the formulated patches showed optimum stability with no obvious physicochemical changes [41].

## 5. Conclusions

The results of the current study indicate that methotrexate, having EC/HPMC polymers at different concentrations, has the best and excellent patch-forming abilities. All formulated patches (F1–F9) were evaluated, and the F5 formulation exhibited the best in vitro drug release pattern and ex vivo drug permeation ability, having the highest deposition of methotrexate compared to other formulated patches. This greater retention on the deeper layer of the skin is significant for targeting psoriasis because this chronic ailment prevails in the epidermis and dermis layers. Thus, F5 showed the best potential for transdermal drug delivery. The controlled and slow release of the drug showed that the F5 formulation is suitable for transdermal patches. FTIR studies showed no interaction between the drug (methotrexate) and polymers (EC/HPMC) used. Methotrexate was distributed uniformly in the formulated patches and was of an amorphous nature.

## Figures and Tables

**Table 1:** Composition of methotrexate transdermal patches.

Batch	Amount of MTX (mg)	Total Amount of Polymers			Plasticizer PEG-400 (%)	Amount of Solvents (*v*/*v*) mL	
		EC (mg)	HPMC (mg)	Combination EC/HPMC		Dichloromethane	Ethanol
F1 (Control)	5	100	100	1:1	25	20	20
F2	5	100	200	1:2	25	20	20
F3	5	100	300	1:3	25	20	20
F4	5	100	400	1:4	25	20	20
F5	5	100	500	1:5	25	20	20
F6	5	200	100	2:1	25	20	20
F7	5	300	100	3:1	25	20	20
F8	5	400	100	4:1	25	20	20
F9	5	500	100	5:1	25	20	20

**Table 2:** Characterization of MTX-loaded transdermal patches (F1–F9). Data were expressed as mean ± SD, *n* = 3.

Formulation Code	Characteristics							
	Thickness (mm)	Weight Uniformity (mg)	% Moisture Absorbance	% Moisture Loss	% Drug Content	Water Transmission Rate	Folding Endurance	Tensile Strength, Kg/cm^2^
F1	0.51 ± 0.03	73.86 ± 0.05	9.25 ± 1.62	6.28 ± 0.85	97.17 ± 3.21	3.58 ± 0.23	83 ± 2.03	10.43 ± 0.71
F2	0.52 ± 0.02	74.37 ± 0.03	10.81 ± 1.12	7.31 ± 0.21	99.13 ± 2.34	3.77 ± 0.65	92 ± 1.21	9.36 ± 0.83
F3	0.54 ± 0.07	76.55 ± 0.08	11.36 ± 1.32	7.82 ± 0.38	96.97 ± 2.24	3.89 ± 0.34	78 ± 3.24	11.35 ± 0.85
F4	0.55 ± 0.04	77.15 ± 0.05	11.56 ± 0.73	8.25 ± 0.22	97.11 ± 3.23	3.97 ± 0.54	86 ± 2.32	12.75 ± 0.72
F5	0.56 ± 0.04	78.67 ± 0.04	12.79 ± 1.46	8.94 ± 0.62	95.42 ± 2.23	4.23 ± 0.37	89 ± 2.54	10.93 ± 0.76
F6	0.50 ± 0.05	72.75 ± 0.08	8.45 ± 1.22	6.22 ± 0.15	99.54 ± 2.56	4.13 ± 0.41	64 ± 4.62	9.45 ± 0.81
F7	0.51 ± 0.02	73.57 ± 0.04	8.99 ± 1.44	6.58 ± 0.45	97.25 ± 3.43	3.93 ± 0.18	58 ± 5.12	12.34 ± 0.77
F8	0.52 ± 0.04	73.48 ± 0.08	9.67 ± 0.52	7.10 ± 0.23	98.64 ± 1.65	3.69 ± 0.32	52 ± 5.32	10.87 ± 0.83
F9	0.53 ± 0.09	74.86 ± 0.04	10.24 ± 0.97	7.31 ± 0.57	97.67 ± 4.36	3.84 ± 0.69	56 ± 5.35	11.66 ± 0.74

**Table 3:** Stability studies of MTX-loaded transdermal patches (F1–F9), Data were expressed as mean ± SD, *n* = 3. Significant compared to formalin (*p* < 0.05).

Evaluation Parameters	F. Code	30 Days	60 Days	90 Days	120 Days	150 Days	180 Days
Drug content (%)	F1	97.17 ± 3.21	97.02 ± 1.99	96.89 ± 1.11	96.72 ± 3.22	96.61 ± 1.21	96.55 ± 2.11
	F2	99.13 ± 2.01	99.04 ± 1.65	98.85 ± 1.28	98.77 ± 2.14	98.58 ± 3.11	98.49 ± 2.01
	F3	96.97 ± 2.11	96.86 ± 2.56	96.71 ± 1.32	96.59 ± 2.14	96.42 ± 3.01	96.32 ± 2.46
	F4	97.11 ± 3.10	96.89 ± 2.35	96.73 ± 2.12	96.64 ± 2.98	96.59 ± 2.76	96.47 ± 3.23
	F5	95.42 ± 1.23	95.33 ± 2.27	95.09 ± 2.32	94.89 ± 2.65	94.76 ± 2.35	94.66 ± 2.87
	F6	98.92 ± 2.11	98.14 ± 3.11	97.76 ± 2.46	96.56 ± 2.45	95.78 ± 2.26	95.23 ± 3.43
	F7	99.19 ± 3.01	98.89 ± 1.02	97.93 ± 2.75	97.45 ± 3.46	96.69 ± 2.33	96.21 ± 3.10
	F8	97.25 ± 2.76	96.87 ± 3.15	95.87 ± 2.46	95.22 ± 3.25	94.76 ± 3.11	94.32 ± 2.33
	F9	96.63 ± 1.90	96.34 ± 2.32	95.82 ± 3.56	95.25 ± 2.31	94.78 ± 2.10	94.21 ± 2.19
Appearance	F1	No change	No change	No change	No change	No change	No change
	F2						
	F3						
	F4						
	F5						
	F6						
	F7						
	F8						
	F9						

**Table 4:** Results of skin irritation on rabbits. Data were expressed as mean ± SD, *n* = 3. Significant compared to formalin (*p* < 0.05).

Rabbit Groups	Visual Observation	
	Erythema	Edema
	Result of Skin Irritation Studies	
Control	0.00 ± 0.00	0.00 ± 0.00
Adhesive tape	0.46 ± 0.62	1.02 ± 0.19
Blank patch	1.12 ± 0.46	1.06 ± 0.31
F1 Patch	0.83 ± 0.28	1.25 ± 0.16
F2 Patch	0.93 ± 0.67	1.08 ± 0.24
F3 Patch	1.03 ± 0.82	0.83 ± 0.69
F4 Patch	1.25 ± 0.71	1.12 ± 0.33
F5 Patch	0.37 ± 0.54	0.72 ± 0.26
F6 Patch	0.85 ± 0.43	1.05 ± 0.16
F7 Patch	0.94 ± 0.83	1.22 ± 0.35
F8 Patch	1.04 ± 0.74	1.06 ± 0.66
F9 Patch	0.98 ± 0.64	0.86 ± 0.27
Formalin	3.05 ± 0.23	3.21 ± 0.51

**Table 5:** Drug release kinetics (F1–F9), Data were expressed as mean ± SD, *n* = 3.

Formulations	K ± SO	R^2^	N	
F1	0.001 ± 2.8337	0.943	0.307	Fickian diffusion
F2	0.001 ± 5.0820	0.949	0.317	Fickian diffusion
F3	0.002 ± 0.0001	0.940	0.342	Fickian diffusion
F4	0.001 ± 0.0002	0.941	0.345	Fickian diffusion
F5	0.001 ± 0.0005	0.974	0.501	Anomalous non-Fickian diffusion
F6	0.001 ± 0.0001	0.929	0.301	Fickian diffusion
F7	0.001 ± 0.0001	0.924	0.301	Fickian diffusion
F8	0.002 ± 0.0003	0.933	0.303	Fickian diffusion
F9	0.001 ± 0.0006	0.967	0.303	Fickian diffusion

## Data Availability

Not applicable.

## References

[1] Munguía-Calzada P., Drake-Monfort M., Armesto S., Reguero-del Cura L., López-Sundh A.E., González-López M.A. (2021). Psoriasis flare after influenza vaccination in COVID-19 era: A report of four cases from a single center. Dermatol. Ther..

[2] Mehrmal S., Uppal P., Nedley N., Giesey R.L., Delost G.R. (2021). The global, regional, and national burden of psoriasis in 195 countries and territories, 1990 to 2017: A systematic analysis from the Global Burden of Disease Study 2017. J. Am. Acad. Dermatol..

[3] Gottlieb A.B., Merola J.F. (2021). Axial psoriatic arthritis: An update for dermatologists. J. Am. Acad. Dermatol..

[4] Dabholkar N., Rapalli V.K., Singhvi G. (2021). Potential herbal constituents for psoriasis treatment as protective and effective therapy. Phytother. Res..

[5] Zhang B., Lai R.C., Sim W.K., Choo A.B.H., Lane E.B., Lim S.K. (2021). Topical application of mesenchymal stem cell exosomes alleviates the imiquimod induced psoriasis-like inflammation. Int. J. Mol. Sci..

[6] Elmets C.A., Korman N.J., Prater E.F., Wong E.B., Rupani R.N., Kivelevitch D., Armstrong A.W., Connor C., Cordoro K.M., Davis D.M. (2021). Joint AAD–NPF Guidelines of care for the management and treatment of psoriasis with topical therapy and alternative medicine modalities for psoriasis severity measures. J. Am. Acad. Dermatol..

[7] Sudhakar K., Fuloria S., Subramaniyan V., Sathasivam K.V., Azad A.K., Swain S.S., Sekar M., Karupiah S., Porwal O., Sahoo A. (2021). Ultraflexible Liposome Nanocargo as a Dermal and Transdermal Drug Delivery System. Nanomaterials.

[8] Dehshahri A., Kumar A., Madamsetty V.S., Uzieliene I., Tavakol S., Azedi F., Fekri H.S., Zarrabi A., Mohammadinejad R., Thakur V.K. (2021). New horizons in hydrogels for methotrexate delivery. Gels.

[9] Nam S., Mooney D. (2021). Polymeric tissue adhesives. Chemical Reviews. Chem. Rev..

[10] Ezhilarasan D. (2021). Hepatotoxic potentials of methotrexate: Understanding the possible toxicological molecular mechanisms. Toxicology.

[11] Biswasroy P., Pradhan D., Kar B., Ghosh G., Rath G. (2021). Recent Advancement in Topical Nanocarriers for the Treatment of Psoriasis. AAPS PharmSciTech.

[12] Giri B.R., Kim J.S., Park J.H., Jin S.G., Kim K.S., Choi H.G., Kim D.W. (2021). Improved Bioavailability and High Photostability of Methotrexate by Spray-Dried Surface-Attached Solid Dispersion with an Aqueous Medium. Pharmaceutics.

[13] Khan A., Qadir A., Ali F., Aqil M. (2021). Phytoconstituents loaded based nanomedicines for the management of psoriasis. J. Drug Deliv. Sci. Technol..

[14] Imtiaz M.S., Shoaib M.H., Yousuf R.I., Ali F.R., Saleem M.T., Khan M.Z., Sikandar M. (2021). Formulation development and evaluation of drug-in-adhesive-type transdermal patch of metoclopramide HCl. Polym. Bull..

[15] Patel D., Chaudhary S.A., Parmar B., Bhura N. (2012). Transdermal drug delivery system: A review. Pharma Innov..

[16] Bernardes M.T.C.P., Agostini S.B.N., Pereira G.R., da Silva L.P., da Silva J.B., Bruschi M.L., Novaes R.D., Carvalho F.C. (2021). Preclinical study of methotrexate-based hydrogels versus surfactant based liquid crystal systems on psoriasis treatment. Eur. J. Pharm. Sci..

[17] Malviya R., Sundram S., Fuloria S., Subramaniyan V., Sathasivam K.V., Azad A.K., Sekar M., Kumar D.H., Chakravarthi S., Porwal O. (2021). Evaluation and Characterization of Tamarind Gum Polysaccharide: The Biopolymer. Polymers.

[18] Zaidul I.S., Fahim T.K., Sahena F., Azad A.K., Rashid M.A., Hossain M.S. (2020). Dataset on applying HPMC polymer to improve encapsulation efficiency and stability of the fish oil: In vitro evaluation. Data Brief.

[19] Hu M., Yang J., Xu J. (2021). Structural and biological investigation of chitosan/hyaluronic acid with silanized-hydroxypropyl methylcellulose as an injectable reinforced interpenetrating network hydrogel for cartilage tissue engineering. Drug Deliv..

[20] Rekhi G.S., Jambhekar S.S. (1995). Ethylcellulose-a polymer review. Drug Dev. Ind. Pharm..

[21] Wasilewska K., Winnicka K. (2019). Ethylcellulose–a pharmaceutical excipient with multidirectional application in drug dosage forms development. Materials.

[22] Kharia A., Singhai A.K., Gilhotra R. (2020). Formualtion and evalaution of transdermal patch for the treatment of inflammation. J. Pharm. Sci. Res..

[23] Yang X., Tang Y., Wang M., Wang Y., Wang W., Pang M., Xu Y. (2021). Co-delivery of methotrexate and nicotinamide by cerosomes for topical psoriasis treatment with enhanced efficacy. Int. J. Pharm..

[24] Ullah W., Nawaz A., Akhlaq M., Shah K.U., Latif M.S., Alfatama M. (2021). Transdermal delivery of gatifloxacin carboxymethyl cellulose-based patches: Preparation and characterization. J. Drug Deliv. Sci. Technol..

[25] Sahu K., Pathan S., Khatri K., Upmanyu N., Shilpi S. (2021). Development, characterization, in vitro and ex vivo evaluation of antiemetic transdermal patches of ondansetron hydrochloride and dexamethasone. GSC Biol. Pharm. Sci..

[26] Jan S.U., Gul R., Jalaludin S. (2020). Formulation and evaluation of transdermal patches of pseudoephedrine HCL. Int. J. Pharm..

[27] Kulkarni S. (2019). Formulation and evaluation of transdermal patch for atomoxetine hydrochloride. J. Drug Deliv. Ther..

[28] Sakhare A.D., Biyani K.R., Sudke S.G. (2019). Design and Evaluation of Transdermal Patches of Carvedilol. J. Curr. Pharm. Res..

[29] Oxley J.A., Ellis C.F., McBride E.A., McCormick W.D. (2019). A survey of rabbit handling methods within the United Kingdom and the Republic of Ireland. J. Appl. Anim. Welf. Sci..

[30] Ramadon D., McCrudden M.T., Courtenay A.J., Donnelly R.F. (2021). Enhancement strategies for transdermal drug delivery systems: Current trends and applications. Drug Deliv..

[31] Azad A.K., Al-Mahmood S.M., Kennedy J.F., Chatterjee B., Bera H. (2021). Electro-hydrodynamic assisted synthesis of lecithin-stabilized peppermint oil-loaded alginate microbeads for intestinal drug delivery. Int. J. Biol. Macromol..

[32] Bera H., Abbasi Y.F., Gajbhiye V., Liew K.F., Kumar P., Tambe P., Azad A.K., Cun D., Yang M. (2020). Carboxymethyl fenugreek galactomannan-g-poly (N-isopropylacrylamide-co-N, N′-methylene-bis-acrylamide)-clay based pH/temperature-responsive nanocomposites as drug-carriers. Mater. Sci. Eng. C.

[33] Rashid S.A., Bashir S., Ullah H., Ullah Shah K., Khan D.H., Shah P.A., Danish M.Z., Khan M.H., Mahmood S., Sohaib M. (2021). Development, characterization and optimization of methotrexate-olive oil nano-emulsion for topical application. Pak. J. Pharm. Sci..

[34] Sivasankarapillai V.S., Das S.S., Sabir F., Sundaramahalingam M.A., Colmenares J.C., Prasannakumar S., Rajan M., Rahdar A., Kyzas G.Z. (2021). Progress in natural polymer engineered biomaterials for transdermal drug delivery systems. Mater. Today Chem..

[35] Yadav K., Soni A., Singh D., Singh M.R. (2021). Polymers in topical delivery of anti-psoriatic medications and other topical agents in overcoming the barriers of conventional treatment strategies. Prog. Biomater..

[36] Latha A.V.S., Ravikiran T.N., Kumar J.N. (2019). Formulation, Optimization and Evaluation of Glibenclamide Transdermal Patches by using chitosan Polymer. Asian J. Pharm. Technol..

[37] Pünnel L.C., Lunter D.J. (2021). Film-forming systems for dermal drug delivery. Pharmaceutics.

[38] Azad A.K., Al-Mahmood S.M., Chatterjee B., Wan Sulaiman W.M., Elsayed T.M., Doolaanea A.A. (2020). Encapsulation of black seed oil in alginate beads as a ph-sensitive carrier for intestine-targeted drug delivery: In vitro, in vivo and ex vivo study. Pharmaceutics.

[39] Altun E., Yuca E., Ekren N., Kalaskar D.M., Ficai D., Dolete G., Ficai A., Gunduz O. (2021). Kinetic Release Studies of Antibiotic Patches for Local Transdermal Delivery. Pharmaceutics.

[40] Haroon M., Batool S., Asif S., Hashmi F., Ullah S. (2021). Combination of Methotrexate and Leflunomide Is Safe and Has Good Drug Retention Among Patients with Psoriatic Arthritis. J. Rheumatol..

[41] Sabir F., Qindeel M., Rehman A.U., Ahmad N.M., Khan G.M., Csoka I., Ahmed N. (2021). An efficient approach for development and optimisation of curcumin-loaded solid lipid nanoparticles’ patch for transdermal delivery. J. Microencapsul..

